# Generalized Fringe-to-Phase Framework for Single-Shot 3D Reconstruction Integrating Structured Light with Deep Learning

**DOI:** 10.3390/s23094209

**Published:** 2023-04-23

**Authors:** Andrew-Hieu Nguyen, Khanh L. Ly, Van Khanh Lam, Zhaoyang Wang

**Affiliations:** 1 Department of Mechanical Engineering, The Catholic University of America, Washington, DC 20064, USA; hieu.nguyen@nih.gov; 2 Neuroimaging Research Branch, National Institute on Drug Abuse, National Institutes of Health, Baltimore, MD 21224, USA; 3 Department of Biomedical Engineering, The Catholic University of America, Washington, DC 20064, USA; 4 Sheikh Zayed Institute for Pediatric Surgical Innovation, Children’s National Hospital, Washington, DC 20012, USA

**Keywords:** three-dimensional image acquisition, three-dimensional sensing, single-shot imaging, fringe-to-phase transformation, convolutional neural network, deep learning

## Abstract

Three-dimensional (3D) shape acquisition of objects from a single-shot image has been highly demanded by numerous applications in many fields, such as medical imaging, robotic navigation, virtual reality, and product in-line inspection. This paper presents a robust 3D shape reconstruction approach integrating a structured-light technique with a deep learning-based artificial neural network. The proposed approach employs a single-input dual-output network capable of transforming a single structured-light image into two intermediate outputs of multiple phase-shifted fringe patterns and a coarse phase map, through which the unwrapped true phase distributions containing the depth information of the imaging target can be accurately determined for subsequent 3D reconstruction process. A conventional fringe projection technique is employed to prepare the ground-truth training labels, and part of its classic algorithm is adopted to preserve the accuracy of the 3D reconstruction. Numerous experiments have been conducted to assess the proposed technique, and its robustness makes it a promising and much-needed tool for scientific research and engineering applications.

## 1. Introduction

In recent years, three-dimensional (3D) reconstruction has been widely adopted in numerous fields such as medical imaging, robotic navigation, palletization, virtual reality, 3D animation modeling, and product in-line inspection [1,2,3,4]. Generally speaking, 3D reconstruction is a process of creating the 3D geometric shape and appearance of a target, typically from single-view or multiple-view two-dimensional (2D) images of the target. It can be classified into passive and active 3D reconstructions [5,6,7]. The passive approach does not interfere with the target, instead recording the radiance reflected or emitted by its surface. The passive technique requires only imaging components, so it is easy to implement with respect to hardware. Much work has been accomplished in the past many years to increase the speed of the passive 3D reconstruction; nevertheless, the acquired 3D representation tends to have low accuracy if the target lacks sufficient texture variations on its surface [8]. On the other hand, active 3D reconstruction involves using radiation (e.g., laser or structured light) to interfere with the target. It uses relatively more complex hardware components but is often capable of providing 3D shape results with higher accuracy than the passive counterpart, particularly for regions without texture. This paper focuses on the study of the active approach.

Commonly used sensing methods in active 3D reconstruction include laser scanning, time-of-flight (ToF), and structured-light methods [9,10,11]. The laser scanning and ToF techniques are similar, and they measure the 3D profiles of a target by calculating distances traveled by the light. The two methods have gained considerable attention in recent years because of their capabilities of measuring long distances and generating 3D representations at real-time speed (e.g., 30 Hz), which satisfy the sensing requirements in autonomous vehicles and virtual/augmented reality [12,13,14]. A drawback of the techniques is their relatively low accuracy. In addition, the ToF techniques usually use infrared light, which is often interfered with by the background lights and reflections. Such interference hinders its popularity in more sophisticated applications [15]. The structured-light system typically uses a projector to project structured patterns onto a target. The patterns are distorted by the geometric shape of the target surface, and they are then captured for 3D shape reconstruction. The structured-light techniques fall into two categories according to the input: single-shot and multi-shot approaches [16]. Compared with the multi-shot methods, the single-shot ones are faster and can be used for real-time 3D reconstruction. With recent developments, the accuracy of the single-shot techniques has been notably improved [17]. For instance, Fernandez developed a one-shot dense point reconstruction technique integrated with an absolute coding phase-unwrapping algorithm, which provides a dynamic and highly accurate phase map for depth reconstruction [18]. Later, Moreno et al. proposed an approach to estimate the coordinates of the calibration points in the projector image plane using local homographs, through which the acquisition time can be substantially reduced while enhancing the resolution of the acquired 3D shapes [19]. Jensen and colleagues used a differentiable rendering technique to directly compute the surface mesh without an intermediate point cloud, which shortens the computation time and improves the accuracy, especially for objects with sharp features [20]. Recently, Tran et al. built a structured-light RGB-D camera system with a gray-code coding scheme to produce high-quality 3D reconstruction in a real-world environment [21].

Over the past few years, deep learning has gained significant interest in computer vision thanks to its superior modeling and computational capabilities. Specifically, deep learning has been applied to many complicated computer vision tasks such as image classification, object detection, face recognition, and human pose estimation [22,23,24,25]. With unique feature learning capability and extreme computational power, convolutional neural network (CNN) can be utilized for various tasks based on given training datasets. In contrast, unsupervised learning features of deep belief networks, deep Boltzmann machines, and stacked autoencoders can remove the need for labeled datasets [26]. Additionally, deep learning can improve the accuracy of challenging tasks in computer vision while requiring less expert analysis and fine-tuning using trained neural networks compared with the traditional computer vision techniques [27]. With no exception, in 3D reconstruction, deep learning has also been widely employed in numerous studies [28,29]. Recently, Zhang et al. proposed a RealPoint3D network comprising an encoder, a 2D–3D fusion module, and a decoder to reconstruct fine-grained 3D representations from a single image. The 2D–3D fusion module helps generate detailed results from images with either solid-color or complex backgrounds [30]. Jeught and colleagues constructed a CNN on a large set of simulated height maps with associated deformed fringe patterns, which was then able to predict the 3D height map from unseen input fringe patterns with high accuracy [31].

Deep learning-based approaches have been adopted in optical measurement and experimental mechanics to accomplish several classic tasks such as phase extraction, fringe analysis, interferogram denoising, and deformation determination [32,33,34,35,36,37]. Numerous devoted topics in 3D shape measurement involving deep learning have been introduced in the last few years. Notably, a well-known structured-light technique, fringe projection profilometry (FPP), has been integrated frequently with deep learning methods to prepare accurate 3D shapes of objects as high-quality ground-truth labels. A typical FPP-based 3D imaging technique contains several key steps, such as phase extraction, phase unwrapping, fringe order determination, and depth estimation. Although the deep learning models can be applied at various stages of the FPP technique, the objective of producing a high-accuracy 3D shape remains the same.

The 3D shape reconstruction techniques integrating the FPP-based method with deep learning typically fall into two groups: fringe-to-depth and fringe-to-phase approaches. The former group intends to perform an image-to-image transformation using a neural network model where the input–output pair is a single structured-light image and a corresponding depth map [38,39,40,41,42,43]. Compared with the typical deep learning-based depth estimation techniques from a single image in the computer vision field, the major differences include a structured-light input and a high-quality depth map output [44,45,46]. First, the structured-light illumination applies desired feature patterns for accurate geometric information extraction, particularly in the textureless regions. Second, the ground-truth depth maps produced by the FPP-based 3D imaging technique provide higher accuracy (originated from full-field sub-pixel image matching) than the ones obtained from the RGB-D sensors.

**Figure 1:**
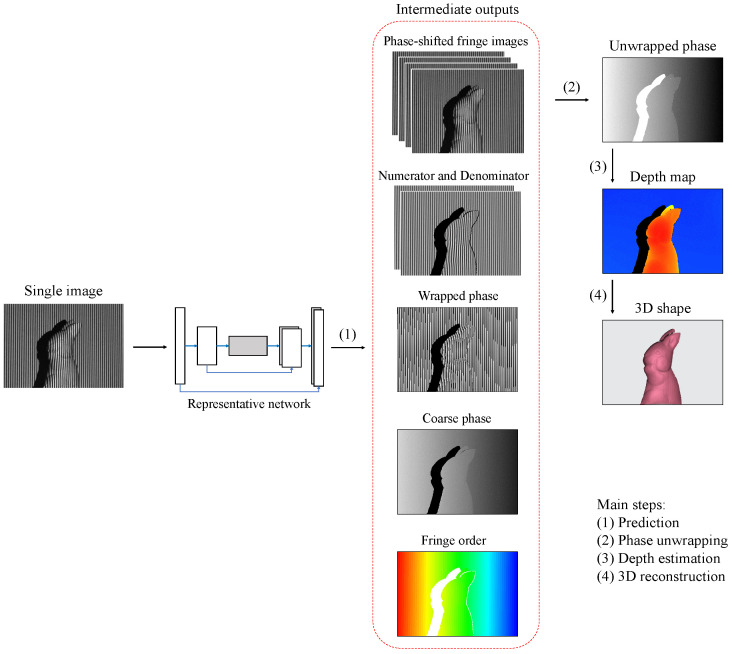
Recent fringe-to-phase approaches integrating structured light with deep learning.

The latter fringe-to-phase group aims to transform fringe pattern(s) into several potential intermediate outputs before determining the unwrapped phase and 3D shape by the conventional technique [47,48,49,50]. Here, *unwrapped* refers to the demodulation of the wrapped phase because conventional algorithms generally yield phase data wrapped in a small value range. Figure 1 demonstrates the pipeline of recent fringe-to-phase approaches. Researchers in [51,52,53,54] developed the pattern-to-pattern schemes where fringe pattern(s) can be converted into multiple phase-shifted fringe patterns. In order to simplify the output and reduce the storage space, the numerator and denominator terms of the arctangent function have been selected as the training output in some neural network-based approaches [55,56,57]. Furthermore, the fringe-pattern input can be transformed directly into the wrapped phase map with a phase range of [−π,π) [58,59,60]. While either phase-shifted fringe patterns, the numerator and denominator, or the wrapped phase map can be obtained via deep learning networks, a map of integer fringe orders is still necessary for the succeeding phase-unwrapping process. The integer fringe orders can be determined using deep learning via an approach such as linear prediction of coarse phase map [61,62] or fringe-order segmentation [63,64,65,66].

Even though the recently developed fringe-to-phase approaches can help achieve high-accuracy 3D shape measurements, there are still many aspects to improve such as eliminating the usage of multiple sub-networks, multiple inputs, reference image, color composite image, and so on. Inspired by the recent fringe-to-phase methods’ advantages, weaknesses, and limitations, this paper presents a novel 3D shape reconstruction technique that transforms a single fringe pattern into two intermediate outputs, phase-shifted fringe patterns and a coarse unwrapped phase, via a single deep learning-based network. Upon successful completion of training, the network can predict outputs of phase-shifted fringe patterns and coarse unwrapped phase to obtain the wrapped phase and integer fringe orders, which further yield a true unwrapped phase for subsequent 3D reconstruction. It is important to note that the full FPP-based technique is only employed for preparing the training dataset, and the 3D reconstruction after prediction just uses part of the FPP-based algorithm. In comparison to the previous fringe-to-depth and fringe-to-phase methods, significant contributions of the proposed technique lie in the following:

1. It requires a single image and a single network. A single network is proposed to transform a single image into four phase-shifted fringe images and an unwrapped coarse phase map;
2. It preserves the accuracy advantage of the classic FPP-based method while eliminating the disadvantage of slow speed originated from capturing multiple phase-shifted fringe patterns;
3. It uses a concise network. Only a single network is used for phase determination instead of multiple sub-networks;
4. It takes a simple image. A single grayscale image is utilized for the training network rather than using a color composite image or an additional reference image;
5. It yields higher accuracy than the image-to-depth approaches.

The rest of the paper is organized as follows. Section 2 depicts the details of the FPP-based technique and the proposed network for phase measurement. Several experiments and relevant assessments are conducted and described in Section 3 to validate the proposed approach. Section 4 includes discussions, and Section 5 gives a brief summary.

## 2. Methodology

The proposed approach uses a supervised deep-learning network to reconstruct the 3D object shape from a single structured-light image. The main task of the network is transforming a single fringe pattern into two intermediate outputs, i.e., phase-shifted sinusoidal fringe patterns and coarse phase distributions, from which the 3D shape can then be reconstructed using a conventional algorithm. In particular, an FPP technique is adopted to prepare ground-truth training labels and part of its algorithm is utilized to accomplish the subsequent 3D reconstruction task after prediction. The employed conventional FPP technique and the proposed network are described in the following subsections.

### 2.1. Fringe Projection Profilometry Technique for 3D Shape Reconstruction

**Figure 2:**
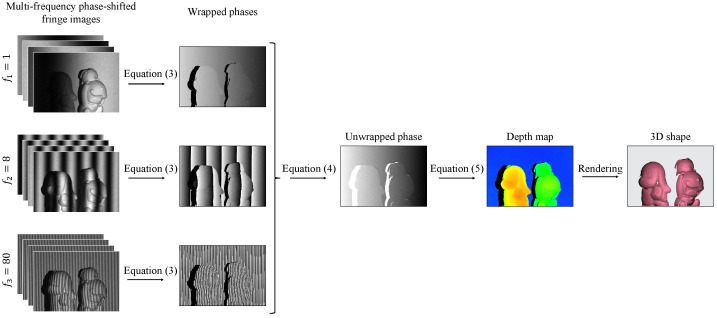
Flowchart of the conventional FPP technique for 3D reconstruction.

The FPP-based 3D imaging system mainly consists of a camera and a projector. During imaging, the projector emits a series of phase-shifted fringe patterns onto the object’s surface, and the synchronous camera captures the distorted fringe patterns in turns. The distorted patterns, in which height or depth information of the target is encoded, are then analyzed to acquire the phase distributions and 3D shapes according to the geometric triangulation. Figure 2 illustrates the pipeline of the classic FPP-based 3D shape reconstruction technique.

The initial fringe patterns are generated following a sinusoidal waveform [67]:

(1) $$I_{n}^{p,(m)}\left(u,v\right)=I_{0}\left[1+\cos\left(\phi^{\left(m\right)}\left(u,v\right)+\delta_{n}\right)\right]$$

where $I^{p}$ is the intensity value of the fringe image at pixel coordinate (u,v); the superscript (m) indicates the *m*th frequency with $m=\left\{1,2,3\right\}$; the subscript *n* denotes the *n*th phase-shifted image with $n=\left\{1,2,3,4\right\}$; $I_{0}$ is the constant amplitude of the fringes and is normally set to $\frac{255}{2}$; ϕ is the fringe phase defined as $\phi^{\left(m\right)}\left(u,v\right)=2\pi f^{\left(m\right)}\frac{u}{W}$, with *f* and *W* being the fringe frequency (i.e., the number of fringes in the whole pattern) and the width of the generated fringe pattern, respectively; δ is the phase-shifting amount with $\delta_{n}=\frac{(n-1)\pi }{2}$.

After being projected onto an object’s surface, the initially evenly spaced fringes follow the surface height or depth profiles and are distorted when seen from the camera view. The distorted fringes have surface profile information encoded into them. Mathematically, the captured fringe patterns can be described as:

(2) $$I_{n}^{\left(m\right)}\left(u,v\right)=I_{a}^{\left(m\right)}\left(u,v\right)+I_{b}^{\left(m\right)}\left(u,v\right)\cos\left[\phi^{\left(m\right)}\left(u,v\right)+\delta_{n}\right]$$

where *I*, $I_{a}$, and $I_{b}$ are the captured fringe intensity, the background intensity, and the amplitude of the intensity modulation at (u,v), respectively. For simplicity, the pixel coordinate (u,v) will be left out in the following equations.

The phase ϕ at each frequency can be retrieved by an inverse trigonometric function as:

(3) $$\phi_{w}^{\left(m\right)}=\mathrm{atan}2\frac{I_{4}^{\left(m\right)}-I_{2}^{\left(m\right)}}{I_{1}^{\left(m\right)}-I_{3}^{\left(m\right)}}$$

In the equation, atan2 denotes the two-argument four-quadrant inverse tangent function; the subscript *w* implies *wrapped* because the output of the arctangent function is in a range of [−π,π), whereas the true or unwrapped phase values have a much broader range. In order to resolve phase ambiguities and retrieve the true phase distributions, we use a multi-frequency phase-shifting (MFPS) scheme that is commonly adopted by the FPP methods. With the MFPS scheme, the true phase distributions are determined by the patterns with the highest frequency, and the wrapped phase values of the lower frequency patterns only serve to obtain the integer fringe orders. Specifically, the phase distributions can be determined by the MFPS scheme as [68,69]:

(4) $$\phi^{\left(m\right)}=\phi_{w}^{\left(m\right)}+INT\left(\frac{\phi^{\left(m-1\right)}\frac{f^{\left(m\right)}}{f^{\left(m-1\right)}}-\phi_{w}^{\left(m\right)}}{2\pi }\right)2\pi$$

where ϕ and $\phi_{w}$ imply the unwrapped and wrapped phase distributions, respectively; INT denotes a function of rounding to the nearest integer; and again, $f^{\left(m\right)}$ represents the *m*th fringe frequency. In this work, the frequencies satisfy $f^{\left(3\right)}>f^{\left(2\right)}>f^{\left(1\right)}$ with $f^{\left(3\right)}=80$, $f^{\left(2\right)}=8$, and $f^{\left(1\right)}=1$, which performs well in practice on dealing with multiple objects and objects with complex shapes. The phase distributions are calculated in the recursive order of $\phi^{\left(1\right)}$, $\phi^{\left(2\right)}$, and $\phi^{\left(3\right)}$, where $\phi^{\left(1\right)}=\phi_{w}^{\left(1\right)}$ is automatically fulfilled for $f^{\left(1\right)}=1$. The desired phase distributions to use for the 3D reconstruction are $\phi =\phi^{\left(3\right)}$.

The out-of-plane height and depth information of the target being imaged is determined by the following model:

(5) $$\begin{array}{cc} z & =\frac{{\langle C,P\rangle }_{F}}{{\langle D,P\rangle }_{F}}\\ C & =\left[\begin{array}{c} 1\;\;c_{1}\;\;c_{2}\;\;c_{3}\;\;\cdots \;\;c_{27}\;\;c_{28}\;\;c_{29} \end{array}\right]\\ D & =\left[\begin{array}{c} d_{0}\;\;d_{1}\;\;d_{2}\;\;d_{3}\;\;\cdots \;\;d_{27}\;\;d_{28}\;\;d_{29} \end{array}\right]\\ P & =\left[\begin{array}{c} 1\;\;u\;\;v\;\;u^{2}\;\;uv\;\;v^{2}\;\;u^{3}\;\;u^{2}v\;\;uv^{2}\;\;v^{3}\;\;u^{4}\;\;u^{3}v\;\;u^{2}v^{2}\;\;uv^{3}\;\;v^{4} \end{array}\right]⊗\left[\begin{array}{c} 1\;\;\phi \end{array}\right] \end{array}$$

where *z* is the physical out-of-plane height or depth of the measurement target, ${\langle \;\rangle }_{F}$ and ⊗ denote the Frobenius inner product and Kronecker product, respectively; $c_{1}-c_{29}$ and $d_{0}-d_{29}$ are 59 parameters. These parameters, together with the camera intrinsic and extrinsic parameters, can be acquired by using a flexible calibration process [70,71].

**Figure 3:**
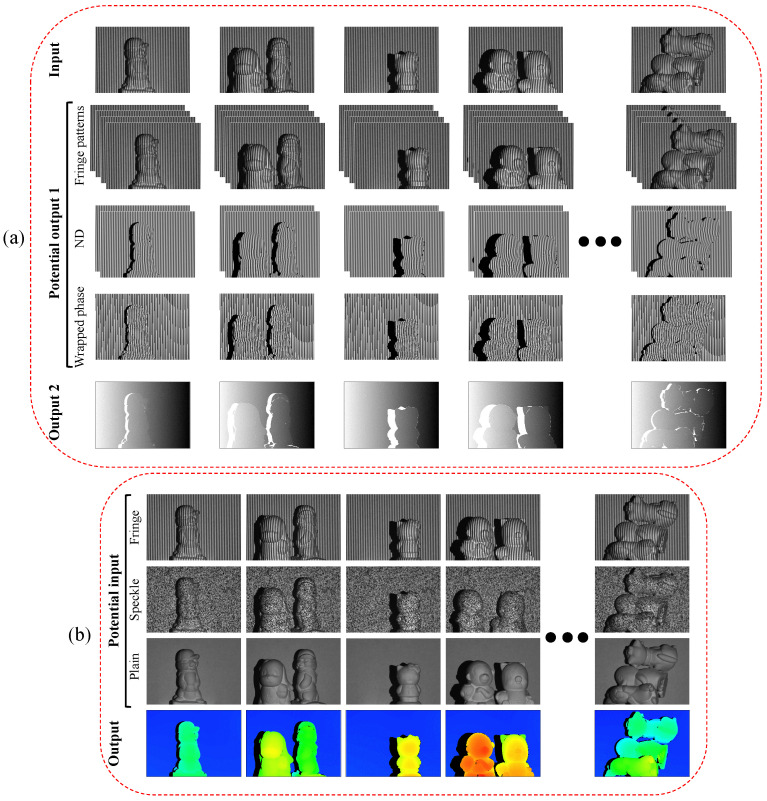
Demonstration of the input–output pairs for (**a**) fringe-to-phase and (**b**) fringe-to-depth approaches.

A dataset of 2048 samples was prepared using the described FPP imaging system, and the sample objects include dozens of sculptures and a number of lab tools. In the data acquisition, the projector sequentially projects 14 pre-generated images, and the camera synchronously captures 14 corresponding images. Specifically, the first 12 images are required by the conventional FPP method, as described previously; they are the four-step phase-shifted fringe images with three frequencies of 1, 8, and 80 fringes per image. Among the corresponding 12 captured fringe images, the four images with the highest fringe frequency serve as the first output labels of the network model. Meanwhile, the unwrapped phase map determined from the 12 capture images by using Equations (3) and (4) serves as the second output label. The input, on the other hand, is one of the aforementioned four images (e.g., the fourth one) with the highest fringe frequency. Therefore, the input to the network is a high-frequency fringe image, and the output includes the input image itself and three associated phase-shifted fringe images as well as a corresponding unwrapped phase map. Furthermore, since the numerator and denominator (ND) terms of the arctangent variable or the wrapped phase (WP) shown in Equation (3) may substitute the phase-shifted fringe images as the network’s first output, they are obtained as well during the FPP processing for comparison purposes. Consequently, three input–output pairs are generated, where the datasets are the same except for the first output. Figure 3a demonstrates a few representative input–output pairs.

Along with the fringe-to-phase datasets, we also prepared a few image-to-depth datasets to compare the proposed method with other relevant deep learning-based techniques. For this reason, two additional projection images, i.e., the 13th and 14th images, are included in the dataset preparation. They include a random speckle pattern image and a uniform white image. In the image-to-depth datasets, the depth map is generated by using the first 12 phase-shifted fringe patterns and Equations (3)–(5), and each of the last three of the 14 captured images, i.e., the high-frequency fringe image, the speckle pattern, and the plain image, acts as an input for an image-to-depth network. Figure 3b displays some exemplars of the image input and depth-map output pairs. The datasets have been made temporarily accessible at [72].

### 2.2. Single-Input Dual-Output Network for Fringe Image Transformation and Phase Retrieval

**Figure 4:**
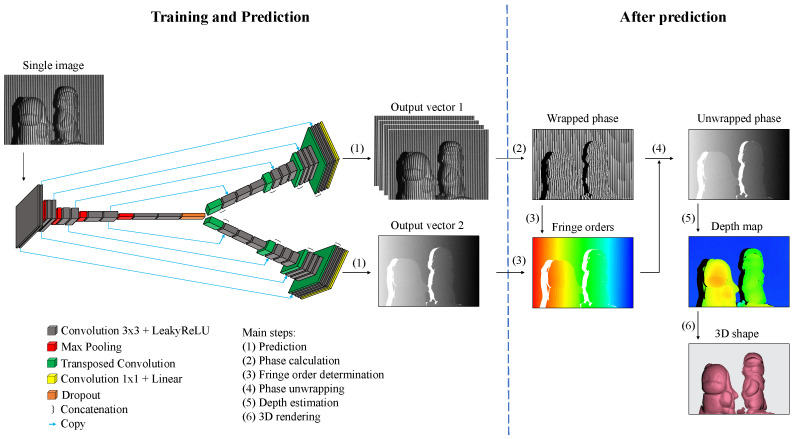
Pipeline of the proposed SIDO network and subsequent 3D reconstruction steps.

Determining the unwrapped phase map in the fringe-to-phase approach is critical despite different intermediate output selections. In the proposed approach, the unwrapped phase map can be determined by using $\phi =\phi_{w}+2\pi k$, which is equivalent to Equation (4). Here, the wrapped phase $\phi_{w}$ is determined from the predicted first output of phase-shifted fringe patterns with Equation (3); the integer fringe order *k* is obtained by the second output (i.e., the predicted unwrapped phase $\phi^{\prime }$) following *k* = INT $\left(\frac{\phi^{\prime }-\phi_{w}}{2\pi }\right)$. It is noteworthy that the predicted $\phi^{\prime }$ is not directly used as the true unwrapped phase for subsequent depth calculation because it is relatively noisy. Figure 4 demonstrates the pipeline of the proposed single-shot 3D reconstruction technique where the first part describes the employment of a deep learning network to predict two intermediate outputs, and the second part explains the subsequent 3D reconstruction with part of a classic FPP algorithm. Since the proposed fringe-to-phase approach transforms a single fringe-pattern image into two outputs, it is called a single-input dual-output (SIDO) network hereafter.

The SIDO network is adapted from the well-known autoencoder-based network, UNet [73]. The SIDO network preserves the prominent concatenation of the UNet but contains two decoder paths instead of one. The left portion of Figure 4 displays the network architecture. In the network, the encoder path and the first decoder path perform a pattern-to-pattern transformation where a single fringe pattern can be converted to four phase-shifted fringe patterns of the same frequency with an even phase-shifting increment of π/2. The second learning path, including the same encoder and a different decoder, is trained to obtain an unwrapped phase map. The encoder path extracts local features from the input with ten convolution layers (a kernel size of 3 × 3) and four max-pooling layers (a window size of 2 × 2). After each max-pooling layer, the resolution of the feature maps is reduced by half, but the filter depth after each pair of convolution layers is doubled. In contrast, each decoder path consists of eight convolution layers and four transposed convolution layers. The input feature maps from the encoder path are enriched to higher resolution while decreasing the filter depths. The sequence of the filter depths in the encoder path is 32, 64, 128, 256, and 512, while the ones in both decoder paths are 256, 128, 64, and 32. In addition, symmetric concatenations between the encoder and decoder paths are employed to maintain the precise feature transformation from the input to the output. In particular, a 1×1 convolution layer with a filter size of 4 is attached to the end of the first decoder path to lead the internal feature maps to the corresponding four phase-shifted fringe images. Similarly, a 1×1 convolution layer (a filter size of 1) is appended after the second decoder path for the unwrapped phase prediction. Since both outputs contain continuous variables, a linear activation function and a common regression loss function, mean-squared error (MSE), are implemented for the training of the proposed fringe-to-phase framework. Importantly, a leaky rectified linear unit (LeakyReLU) function with a negative coefficient of 0.1 is applied over the convolution layers to avoid the zero-gradient problem. Moreover, a dropout function is added between the encoder path and the two decoder paths.

The multidimensional data format of the input, output, and internal hidden layers is a four-dimensional tensor of shape $\left[s,h,w,c\right]$ where *s* denotes the number of data samples; *h* and *w* represent the height and width of the input, output, or feature maps at the sub-scale resolution layer, respectively; *c* is the channel or filter depth. In this work, *c* is set to 1 for the input of a single grayscale image, and *c* for the two outputs are set to 4 and 1, corresponding to the four phase-shifted fringe images and the unwrapped phase map, respectively.

Using the backpropagation process, we trained the network parameters through 400 epochs using a mini-batch size of 1 or an equivalent stochastic gradient descent scheme. Adam optimizer with an initial learning rate of 0.0001 is set for the first 300 epochs. After that, a step decay schedule [74] is adopted to gradually reduce the learning rate for further convergence. Several data augmentation functions (e.g., ZCA whitening, brightness and contrast augmentation) are also adopted to prevent unwanted overfitting. Finally, some typical measures (e.g., MSE, RMSE) with Keras callbacks (e.g., History, ModelCheckpoint) are taken to monitor the history of the training process and save the best convergent model.

## 3. Experiments and Assessments

A series of experiments and analyses have been performed to validate the robustness and practicality of the proposed fringe-to-phase approach. An RVBUST RVC-X mini 3D camera, which offers a desired camera-projector-target triangulation setup, is employed to capture the datasets with a resolution of 640×448 pixels. The reduced resolution comes from directly cropping the full-size images (1920×1200 pixels). The use of reduced resolution is to accommodate the assigned computation resource, which includes multiple GPU nodes in the Biowulf cluster of the High Performance Computing group at the National Institutes of Health to accelerate the training process. The main graphics processing units (GPUs) include 4 × NVIDIA A100 GPUs 80GB VRAM, 4 × NVIDIA V100-SXM2 GPUs 32GB VRAM, and 4 × NVIDIA V100 GPUs 16GB VRAM. Furthermore, Nvidia CUDA Toolkit 11.0.3 and cuDNN v8.0.3 were installed to enhance the performance of the aforementioned units. TensorFlow and Keras, two popular open-source and user-friendly deep learning frameworks and Python libraries, were adopted to construct the network architecture and to use in data predictions and analysis.

### 3.1. Can the Predicted Unwrapped Phase Map Be Used Directly for 3D Reconstruction?

After a successful training process, the achieved network model is ready for an application test. It takes in a fringe-pattern image of the test target as a single input and produces two intermediate outputs: four phase-shifted fringe-pattern images and a coarse unwrapped phase map. As previously described, the obtained fringe patterns serve to calculate the accurate wrapped phase map, which is then applied together with the predicted coarse unwrapped phase to get the integer fringe orders. After that, the refined unwrapped phase distribution can be determined. This procedure is established as an equation as follows:

(6) $$\begin{array}{cc} \phi_{w} & =\mathrm{atan}2\frac{I_{4}^{\left(p\right)}-I_{2}^{\left(p\right)}}{I_{1}^{\left(p\right)}-I_{3}^{\left(p\right)}}\\ \phi & =\phi_{w}+2\pi \cdot \mathrm{INT}\left(\phi^{\left(p\right)}-\phi_{w}\right) \end{array}$$

where $I_{1}^{\left(p\right)}$–$I_{4}^{\left(p\right)}$ indicate the intensities of the network-predicted images and $\phi^{\left(p\right)}$ denotes the network-predicted coarse unwrapped phase.

**Figure 5:**
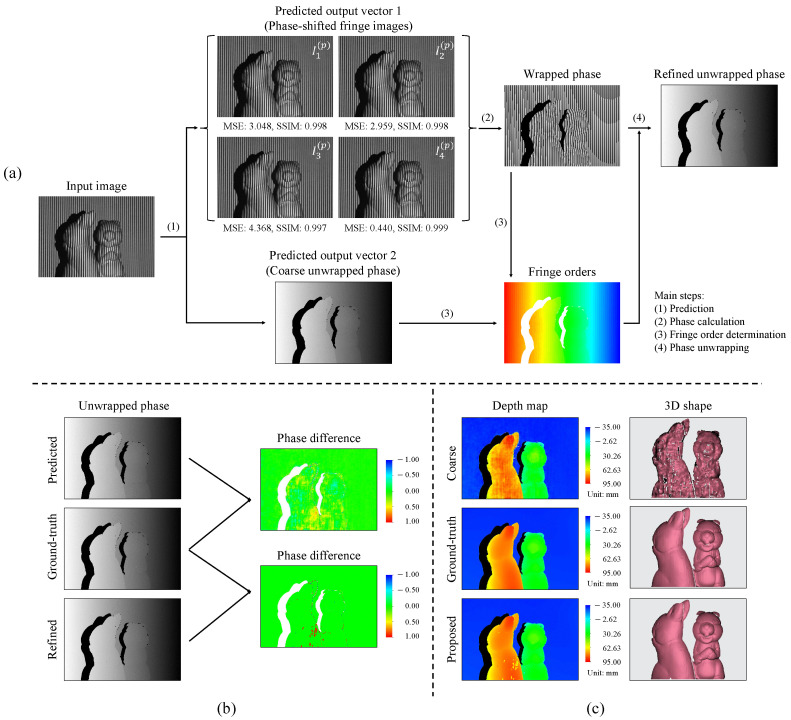
Qualitative and quantitative 3D measurement of a test sample. (**a**) Output prediction and phase determination process; (**b**) phase comparisons; and (**c**) 3D visualizations.

The whole phase determination process using a single grayscale image and a single network is illustrated in Figure 5a. Two types of evaluation metrics, MSE and Structural Similarity Index Measure (SSIM), are employed to assess the performance of the proposed network in the fringe prediction task. Following the quantitative assessment shown in Figure 5a, the metrics reveal that the four predicted fringe patterns are very similar to the ground-truth fringe patterns; particularly, the last one has the smallest MSE error and the highest SSIM. This is expected since the last image ideally should be identical to the input.

As the second output is the predicted unwrapped phase, one question which can arise is the following: can the predicted unwrapped phase be employed directly for the 3D shape reconstruction following Equation (5)? In the extended investigation shown in Figure 5b,c, the predicted unwrapped phase $\phi^{\left(p\right)}$ and the refined phase ϕ are compared. It is clear that the errors in the refined phase map occur in small and limited areas, whereas the errors originating from the predicted unwrapped phase spread out to larger regions. The depth maps and the 3D shapes associated with both evaluated unwrapped phases are reconstructed to further demonstrate their performance. It is evident from the results that the predicted phase map results in an inconsistent depth map and noisy 3D shape, while the refined phase map from the proposed method can produce a high-accuracy 3D shape similar to the ground-truth label.

### 3.2. Full-Surface 360${}^{\circ }$ 3D Reconstruction of a Single Object

**Figure 6:**
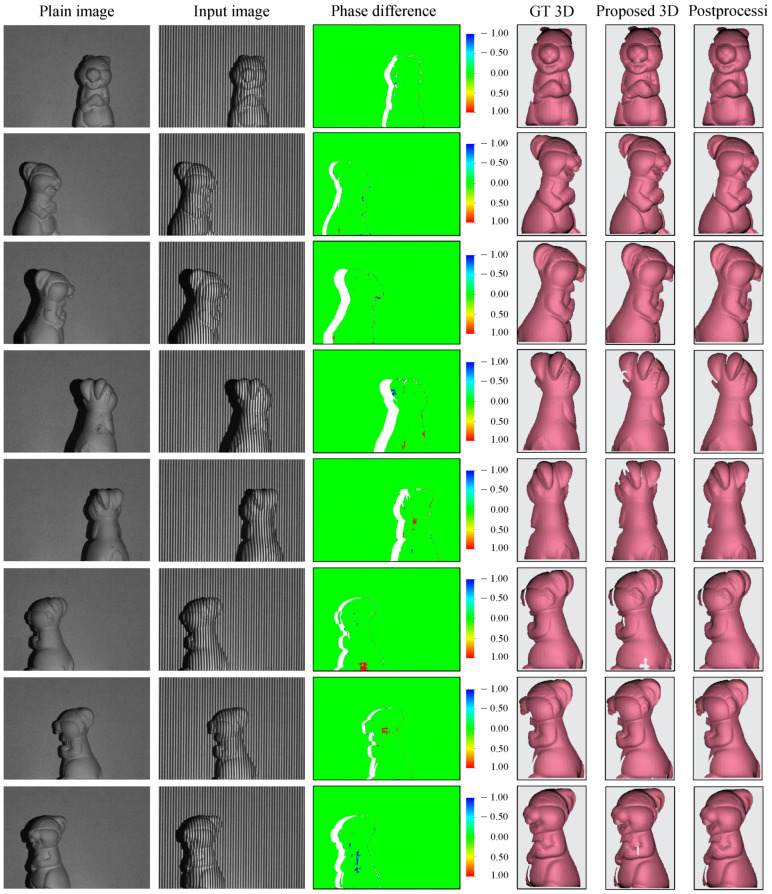
Full-surface $360^{\circ }$ 3D shape reconstruction of an object.

Three-dimensional full-surface reconstruction is a classic yet challenging task in computer vision and relevant fields because it demands high-accuracy 3D point clouds. An acquisition of multi-view 3D surface profiles has been conducted to demonstrate the capability of our proposed technique. In this experiment, an untrained object (i.e., never used in the network training) has been rotated and positioned randomly for capturing. Figure 6 demonstrates eight single-view 3D shape reconstructions of the test object. The first two columns are the plain images and the input images, followed by the phase differences between the ground-truth phase and the refined unwrapped phase distributions. The last three columns display the ground-truth 3D shape, the direct 3D reconstruction of the proposed technique, and the 3D shape after post-processing, respectively. In general, major errors in phase difference are observed along the edges where the fringes could not be illuminated effectively. This results in some holes on the surfaces of the 3D shapes that can be easily erased or fixed in a post-processing step thanks to the accurately reconstructed points surrounding them. The final 3D shapes obtained from our proposed 3D reconstruction are nearly identical to the ground-truth 3D acquired by the conventional temporal-based FPP technique. Finally, a $360^{\circ }$ full-surface 3D image is built after data alignment and stitching (see Supplementary Video S1).

### 3.3. Three-Dimensional Reconstruction of Multiple Objects

**Figure 7:**
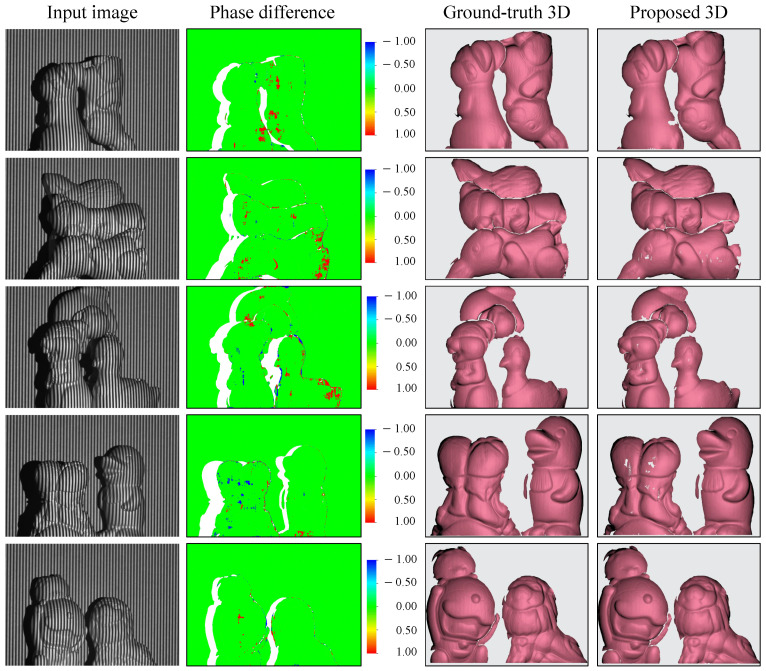
Three-dimensional shape reconstruction of multiple separated objects.

In real-world applications, the target of interest may include multiple separated objects, which typically bring an issue of geometric discontinuity. In this experiment, several untrained objects were grouped and randomly located in the scene to verify the capability of the proposed technique. Figure 7 shows the 3D reconstruction results. It is evident that the SIDO network can generally handle multiple objects well, and the reconstructed 3D surface profiles are close to the ground-truth 3D shapes with sub-millimeter errors. In the meantime, it can be seen that there are more errors than in the case of a single object. This is reasonable since there are more complex edges with multiple separated objects. These errors present as holes or stay close to the edges, and they can be easily fixed or eliminated by post-processing.

### 3.4. Three-Dimensional Reconstruction of Objects with Varied Colors

**Figure 8:**
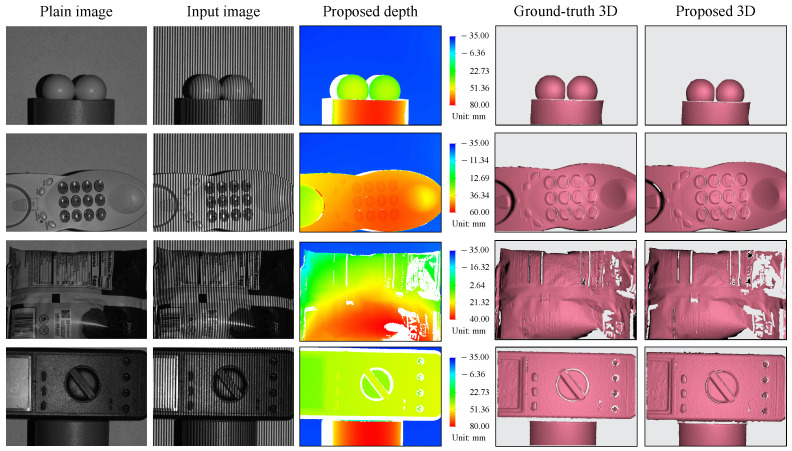
Three-dimensional shape reconstruction of different objects with a variety of colors and contrasts.

To further explore the applicability of the proposed method, we proceed with the testings using a few complex targets with various colors and contrasts. Figure 8 presents the objects and their 3D shape reconstruction results. The first and second columns display the plain images and the input images, respectively, followed by the determined depth maps, the ground-truth 3D shapes, and the reconstructed 3D shapes. Our method can clearly produce high-quality 3D shape reconstructions for complex objects, as shown by a comparison with the ground-truth shapes in the first, second, and fourth rows. It is noted that evident errors emerge in the third object because that object contains intricate textures and more shaded regions, making the 3D reconstruction more challenging even for the conventional technique.

### 3.5. Accuracy Comparison of the Proposed 3D Reconstruction Technique with Other Fringe-to-Phase and Fringe-to-Depth Methods

In recent years, the integration of structured light with deep learning has received ever-increasing attention. To demonstrate the efficacy of the proposed techniques, we attempt to compare the performance of our technique with five other existing state-of-the-art techniques, classified into fringe-to-phase and image-to-depth groups. In the fringe-to-phase group, the second output of unwrapped phase remains unchanged while the first output is replaced with either phase-shifted fringe images (proposed method), numerator and denominator (fringe-to-ND), or wrapped phase map (fringe-to-WP). For the image-to-depth technique, since it is a direct transformation from a grayscale image into a depth map, the depth map is fixed and only the grayscale input image is replaced among the fringe (fringe-to-depth), speckle (speckle-to-depth), and plain images (plain-to-depth). These image-to-depth techniques are trained based on the adopted autoencoder-based network with multi-scale feature fusion [75]. It is important to note that since the primary comparison in this experiment only takes a single grayscale image as input, other techniques that require a composite red-green-blue image [53,54,57,58], multiple images [11,41,51,52,62,64], or a reference image [34,40,55] are not considered. Several error and accuracy metrics commonly used for the evaluation of monocular depth reconstruction are adopted here. The quantitative assessments contain the following four error metrics and three accuracy metrics:

- Absolute relative error (rel): $\frac{1}{n}\sum_{i=1}^{n}\frac{\left|\hat{z_{i}}-z_{i}\right|}{\hat{z_{i}}}$;
- Root-mean-square error (rms): $\sqrt{\frac{1}{n}\sum_{i=1}^{n}{\left(\hat{z_{i}}-z_{i}\right)}^{2}}$;
- Average $log_{10}$ error (log): $\frac{1}{n}\sum_{i=1}^{n}\left|\begin{array}{c} log_{10}\left(\hat{z_{i}}\right)-log_{10}\left(z_{i}\right) \end{array}\right|$;
- Root-mean-square log error (rms log):
  $\sqrt{\frac{1}{n}\sum_{i=1}^{n}\left|\begin{array}{c} log_{10}\left(\hat{z_{i}}\right)-log_{10}\left(z_{i}\right) \end{array}\right|}$;
- Threshold accuracy: $\delta =(\frac{\hat{z_{i}}}{z_{i}},\frac{z_{i}}{\hat{z_{i}}})<thr;$
  $thr\in \left\{\begin{array}{c} 1.25,1.25^{2},1.25^{3} \end{array}\right\}$.

**Figure 9:**
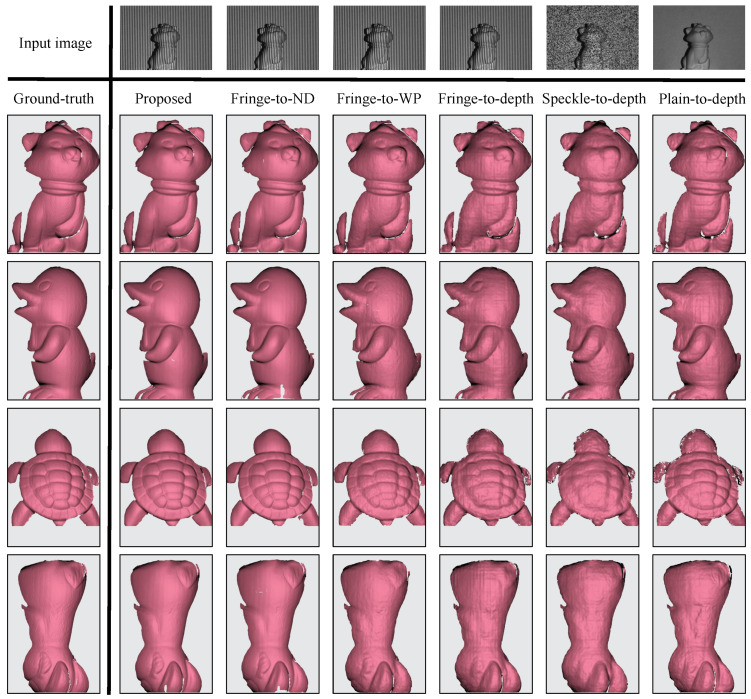
Qualitative comparisons between the proposed and other deep learning-based structured-light techniques.

Figure 9 shows the corresponding 3D reconstructions of the proposed, fringe-to-phase, and image-to-depth techniques. It is clear that the three fringe-to-phase techniques outperform the image-to-depth counterparts because the intermediate results help retain more feature information. It can also be seen that several holes and incorrect shape regions appear in the fringe-to-ND and fringe-to-WP results, which is probably due to the misalignment between predicted outputs and the obtained fringe orders. It is also shown that the speckle pattern is not a desired option for the image-to-depth network [36] since surface textures can be concealed by random pattern (unlike regular fringe pattern). Furthermore, the plain-to-depth technique obtains noisy 3D results despite being capable of yielding discernible textures. Table 1 summarizes the numerical errors and accuracy metrics of the proposed and other techniques. The first three rows from Table 1 reveal that the accuracy performances of the proposed and the fringe-to-ND techniques are similar, while the fringe-to-WP approach offers a slightly lower performance.

## 4. Discussions

This work explores a supervised learning-based approach to intervening in the classic FPP-based technique for single-shot 3D shape reconstruction. The proposed technique uses a single fringe image and a SIDO network to acquire two intermediates for subsequent accurate 3D shape reconstruction. The well-known conventional FPP technique is introduced and implemented to generate high-quality datasets for training the deep-learning network. Compared with many other techniques in the same category, the proposed technique uses only a single grayscale fringe image as input instead of requiring multiple images, a composite RGB image, or a reference image. This makes the proposed technique very appealing for numerous engineering applications where multi-shot capturing is undesired. Such applications typically involves dynamic motions, such as robotic navigation, automatic palletization and material handling in warehouse, in-line inspection of products during manufacturing, virtual/augmented reality, and 3D human body scanning.

Even though the proposed technique closely resembles the well-known classic FPP technique for accurate 3D shape reconstruction, the results can be partially sketchy because of the dependence on the supervised-learning datasets. As the phase distributions depend on not only the actual object profiles but also the geometric setup of the imaging system, the trained model is valid only for the specific system configuration used in dataset generation. If the relative geometric configuration between the camera and projector changes, recapturing new datasets for training is imperative. For this reason, generating synthetic datasets based on the calibration parameters of the actual system for network training can be a favorable solution [38,45]. Nevertheless, further investigation is necessary to make such a network model reliable for real-world applications.

The misalignment between two outputs can create errors in the phase map that can lead to incorrect or invalid height/depth. In addition, the unbalanced intensities of the predicted phase-shifted fringe patterns near the edges can construct observable errors there. Despite that, a post-processing step can easily eliminate the wrong points and readily fill the holes without noticeable issues thanks to the accurate reconstruction of the neighboring points.

The series of experiments conducted have verified the validity and robustness of the proposed method for 3D shape reconstructions. The experiments also demonstrate that the fringe-to-phase methods can yield better 3D shapes than the image-to-depth ones, thanks to the feature-preserving characteristics of the intermediate outputs. Although the fringe-to-phase approach requires extra computation time (typically a few to tens of milliseconds) to calculate the 3D point clouds from the predicted outputs, it is worth the wait since 3D shapes with high-quality texture details can be achieved. In addition, the extra calculation can be accelerated by parallel processing with the TensorFlow framework. It is noticed that using the numerator and denominator (fringe-to-ND) instead of using four phase-shifted fringe images as the first output is feasible to save storage space without perceptibly affecting the final 3D results. Lastly, it is noteworthy that the predicted unwrapped phase can be directly used to reconstruct the 3D shape with evident errors; however, we believe that the results can be considerably improved upon exploring a more suitable network with meticulously tuned learning hyperparameters and network parameters.

## 5. Conclusions

In summary, the paper presents an innovative 3D shape reconstruction technique integrating the classic FPP technique with deep learning. The technique requires only a single concise network, and it takes a single fringe image as the input. An advanced autoencoder-based network inspired by the UNet has been implemented to convert the single input image into two immediate outputs of four phase-shifted fringe patterns and a coarse unwrapped phase map. These outputs can yield a refined accurate unwrapped phase map, which can then be used to determine the depth map and reconstruct the 3D shapes. The key advantage of the proposed technique lies in using a single image for 3D imaging and shape reconstruction, thus, the capturing speed can be maximized; At the same time, using network-predicted multiple images for subsequent calculations helps preserve the high-accuracy nature of the result. Therefore, both fast speed and high accuracy can be achieved for the 3D shape reconstruction, which provides a promising tool in numerous scientific and engineering applications.

## Figures and Tables

**Table 1:** Quantitative performance comparison of different fringe-to-phase and image-to-depth techniques.

Method	Error (Lower Is Better)					Accuracy (Higher Is Better)		
	rel	rms	log	rms log		δ<1.25	$\delta <1.25^{2}$	$\delta <1.25^{3}$
Proposed method	0.004	0.385	0.003	0.050		99.5%	99.8%	99.9%
Fringe-to-ND	0.005	0.392	0.003	0.049		99.5%	99.8%	99.9%
Fringe-to-WP	0.007	0.610	0.004	0.061		99.2%	99.7%	99.8%
Fringe-to-depth	0.023	0.916	0.015	0.119		97.7%	99.1%	99.5%
Speckle-to-depth	0.027	0.923	0.016	0.120		98.0%	99.2%	99.6%
Plain-to-depth	0.031	1.104	0.020	0.134		96.8%	98.9%	99.4%

## Data Availability

The data presented in this study are available on request from the corresponding author.

## Supplementary Materials

The following supporting information can be downloaded at: https://www.mdpi.com/article/10.3390/s23094209/s1, Video S1: $360^{\circ }$ full-surface 3D image of an object.
